# A 23-year-old man with acute lung injury after using a tetrahydrocannabinol-containing vaping device: a case report

**DOI:** 10.1186/s13256-020-02549-9

**Published:** 2021-02-11

**Authors:** Anthony Lucero, Niklas Eriksson, Carli Nichta, Kimberly Sokol

**Affiliations:** Department of Emergency Medicine, Kaweah Delta Medical Center, 400 West Mineral King Avenue, Visalia, CA 93291 USA

**Keywords:** VALI, Vaping, Lung injury, Case report

## Abstract

**Background:**

Vaping-associated lung injury is a newly emerging disease process with the potential for serious health implications and high mortality, even despite the lack of underlying lung disease. We present a case of a young, otherwise healthy patient with tetrahydrocannabinol vaping-associated lung injury.

**Case presentation:**

A 23-year-old Caucasian man with a past history of tetrahydrocannabinol vaping and benzodiazepine and methamphetamine abuse presented to the emergency department of our institution with a complaint of “feeling malnourished” over the past 5 days, along with associated fevers, cough, and vomiting. His past medical, surgical, family, and social histories were significant only for the recent use of marijuana vaping pens. Upon initial presentation, the patient appeared to be in significant respiratory distress. A computed tomographic scan of his chest demonstrated diffuse central predominant interstitial opacities, and he was admitted to the medical intensive care unit, where he was eventually intubated for hypoxic respiratory failure. No other cause of his respiratory failure was found, and it was ultimately believed that the patient had sustained a vaping-associated lung injury.

**Conclusion:**

Tetrahydrocannabinol-containing vaping-associated lung injury is still poorly understood overall and is currently being investigated by the Centers for Disease Control and Prevention. In the meantime, physicians should consider vaping to be a public health emergency. We summarize the appropriate history, physical examination, appropriate workup, and therapies that physicians should be aware of in order to appropriately manage and treat patients presenting with suspected vaping-associated lung injury.

## Introduction

According to the Centers for Disease Control and Prevention (CDC), as of 18 February 2020, there have been 2807 lung injury cases associated with the use of vaping products in the United States [1]. Sixty-eight deaths in 29 states have been confirmed [1]. On the basis of a recent issue of the CDC’s *Morbidity and Mortality Weekly Report*, among 805 cases of vaping-associated lung injury (VALI), patients were mostly young adult males (69% male; 62% aged 18–34 years) [2]. Tetrahydrocannabinol (THC)-containing products were implicated in 79.6% of case reports [2]. The THC-containing products linked to many of these cases have been obtained from unregulated sources, suggesting that there may be an additive that is further contributing to the disease [3]. Recent literature suggests additives such as vitamin E acetate, diacetyl, and methanol may be implicated in lung disease associated with vaping [4, 5]. Cases reported thus far have shown various forms of pneumonitis, including acute eosinophilic pneumonia, organizing pneumonia, lipoid pneumonia, diffuse alveolar hemorrhage, and acute respiratory distress syndrome [6, 7]. The mechanism of injury is not fully understood; however, given the severity of the disease and its association with vaping products, the CDC is currently recommending against the use of vaping products that contain THC [1].

## Patient information

A 23-year-old Caucasian man with a past history of THC vaping and benzodiazepine and methamphetamine abuse presented to the emergency department of our institution with a complaint of “feeling malnourished” over the past 5 days, along with associated fevers, cough, and vomiting. Upon initial presentation, the patient was responding very poorly to questioning and holding his head in his hands due to discomfort. He admitted to using “vape pens” to smoke marijuana daily for “years,” with his last use being the day prior to presentation. Information about where he obtained his THC vaping product was not elicited. The patient also admitted to abusing methamphetamine and alprazolam and to drinking alcohol occasionally, but he denied smoking cigarettes. He did not take any prescribed medications.

## Clinical findings

The patient’s initial vital signs demonstrated a fever of 38.8 °C, tachycardia with a heart rate of 140 beats per minute, tachypnea with a rate of 24 breaths per minute, oxygen saturation of 95% on a 4-L nasal cannula, and a blood pressure of 151/83 mmHg. On physical examination, the patient was tachypneic with coarse rhonchi noted on auscultation of his lung bases.

## Timeline

**Fig. 1:**
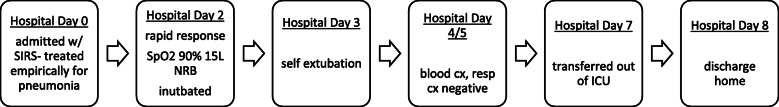
Historical and current timeline of clinical information

Figure 1 is a visual presentation of the timeline.

## Diagnostic assessment

**Fig. 2:**
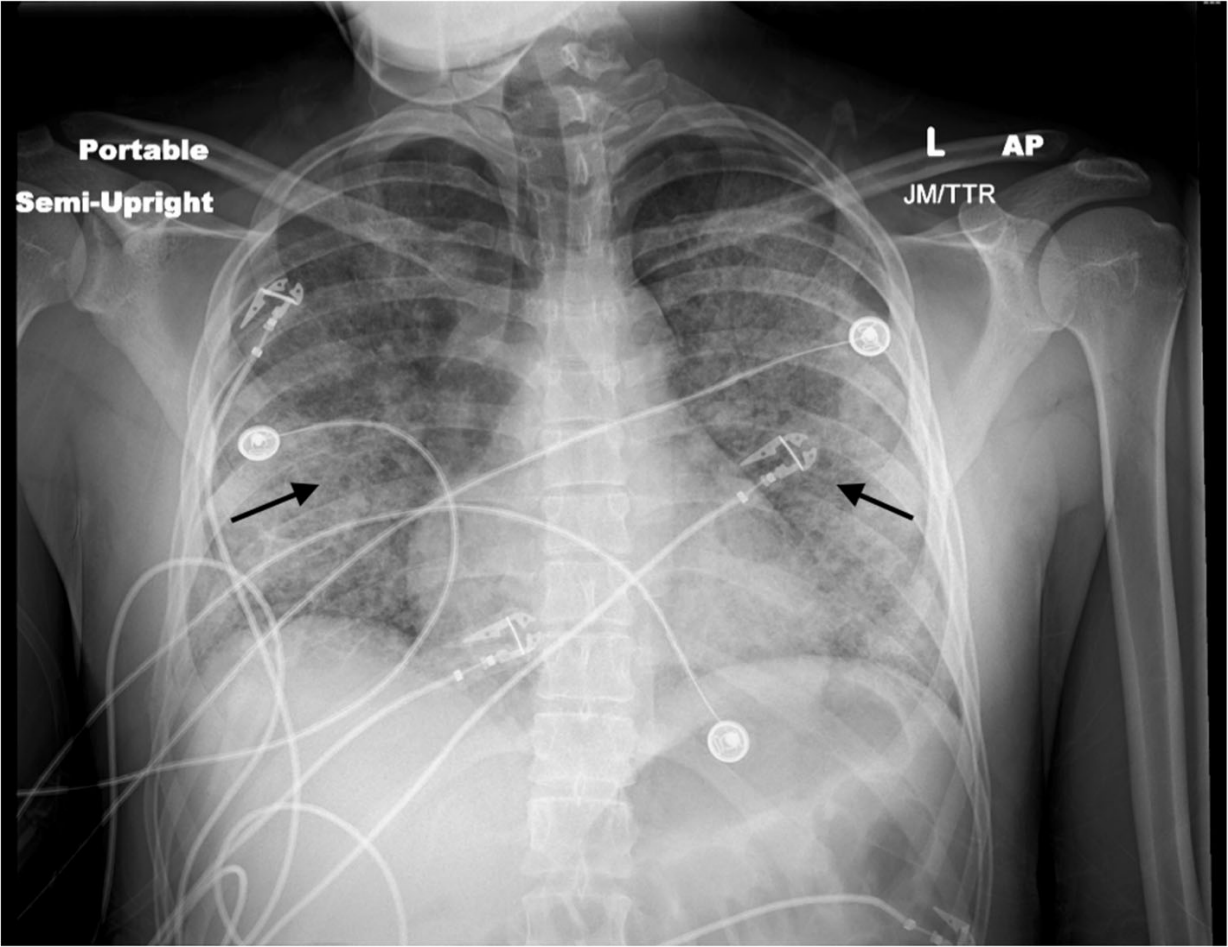
Chest x-ray demonstrating diffuse central predominant interstitial opacities (*black arrows*)

**Fig. 3:**
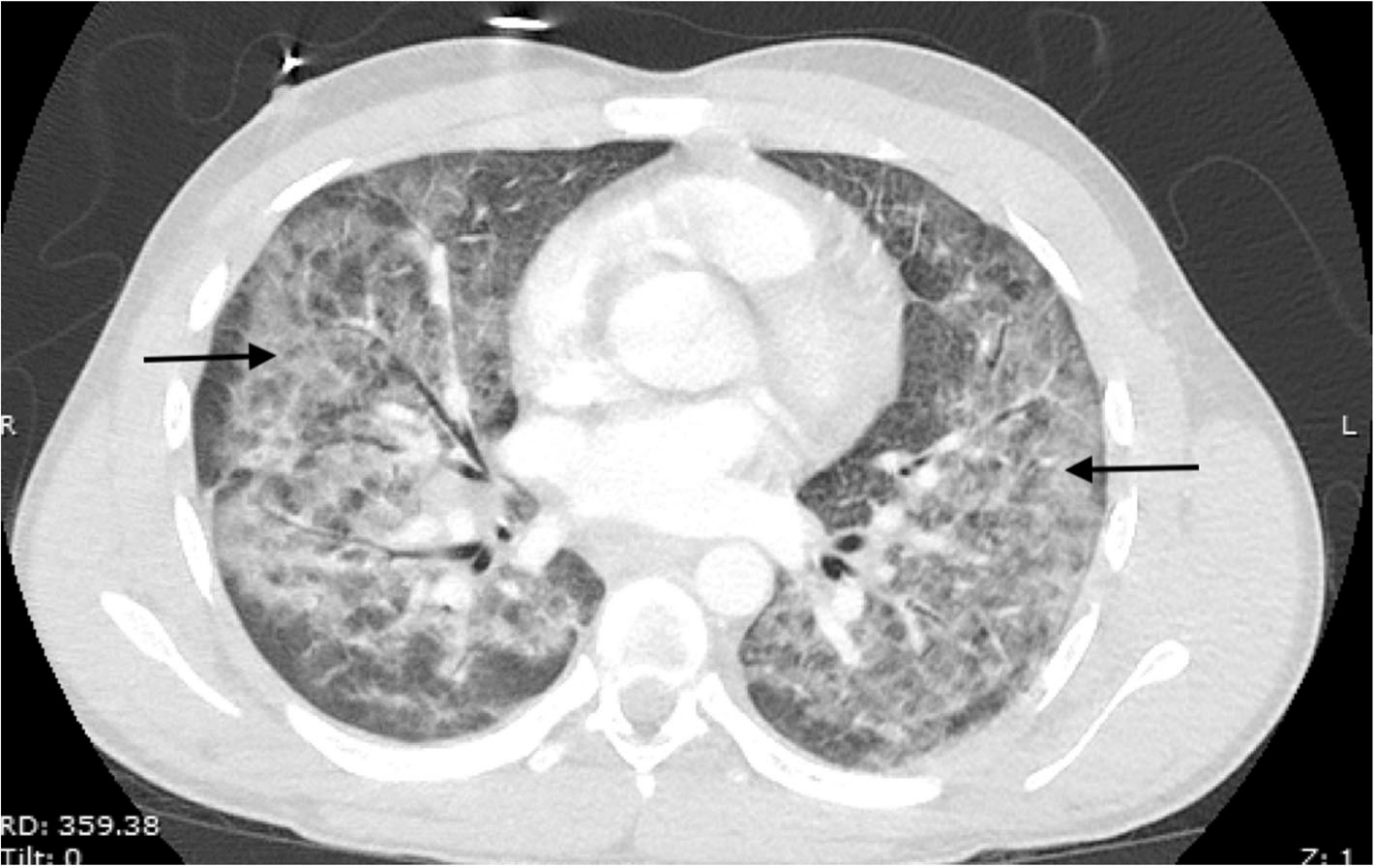
Axial view computed tomographic scan of the chest demonstrating diffuse central predominant interstitial opacities (*black arrows*)

**Fig. 4:**
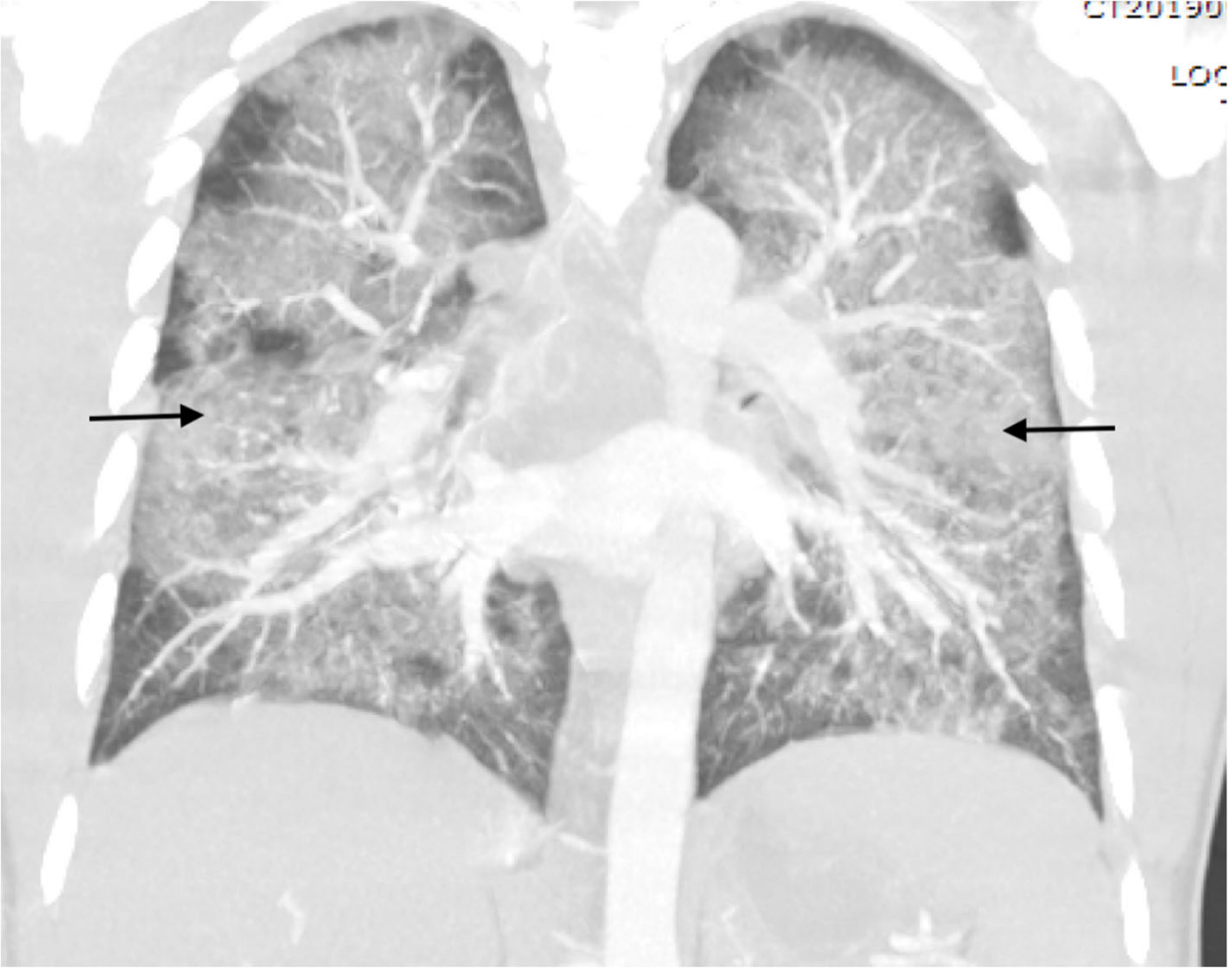
Sagittal view computed tomographic scan further demonstrating diffuse central predominant interstitial opacities (*black arrows*)

Laboratory studies revealed leukocytosis of 17.0 × 10^9^/L with 93% neutrophils and an elevated lactic acid of 1.9 mmol/L. The findings of the patient’s chemistry panel were unremarkable. His urine drug screen result was positive for cannabinoids and benzodiazepines. His erythrocyte sedimentation rate was slightly elevated at 63 mm/hour. His human immunodeficiency virus and hepatitis B and C virus test results were negative. A viral panel was obtained, which showed positive results for rhinovirus and enterovirus, but otherwise negative findings were reported. This patient’s hospitalization occurred before the coronavirus disease 2019 (COVID-19) pandemic; therefore, no COVID-19 testing was available at the time. He had negative test results for antineutrophil antibodies, cytoplasmic and perinuclear antineutrophil cytoplasmic antibodies, and myeloperoxidase. His echocardiogram demonstrated a left ventricular ejection fraction of 64%; otherwise, he had no effusion, no vegetation, or other acute findings. A plain chest x-ray (CXR) (Fig. 2) demonstrated diffuse central predominant interstitial opacities. A computed tomographic (CT) angiogram of the chest was then obtained (Figs. 3 and 4), which confirmed diffuse bilateral interstitial infiltrates, with no evidence of vascular injury or pulmonary embolism.

## Therapeutic interventions

Because the patient remained hypoxic without supplemental oxygen, he was admitted to the medical floor for further management and respiratory care. Intravenous vancomycin and piperacillin-tazobactam were initiated on the basis of concerns for pneumonia.

On hospital day 2, a rapid response was called for worsening tachypnea and hypoxia. The patient was found to have oxygen saturation of only 90% on 15 L via nonrebreather. He was therefore intubated for impending respiratory failure and was transferred to the intensive care unit (ICU). Antibiotic coverage was switched to meropenem, levofloxacin, and aztreonam for further broad-spectrum coverage, given the extent of the bilateral infiltrates and his worsening hypoxia and respiratory status. Fluconazole was also added for fungal coverage until his coccidiomycosis serology was found to be negative, and the medication was subsequently discontinued. Intravenous corticosteroids were then initiated. A bronchoscopy was performed, which revealed scattered small mucosal ulcerations without purulent secretions, masses, or obstruction. On hospital day 3, the patient self-extubated but remained in the ICU for 4 days for further respiratory monitoring. During this time, his oxygen requirement was weaned, and on hospital day 7, he was transferred to the medical floor. He was ultimately discharged to home on hospital day 8 after his respiratory distress and hypoxia had resolved. He was counseled on drug cessation and smoking cessation prior to discharge.

## Follow-up and outcomes

Cytologic studies from the bronchoalveolar lavage (BAL) revealed macrophages, neutrophils, and lymphocytes, with no malignant cells to indicate possible cancer. Our institution’s laboratory did not have the capability of detecting vitamin E acetate, THC, or THC metabolites in the BAL. During our patient’s ICU stay, all of his blood culture and sputum culture results remained negative.

## Discussion

THC-containing vaping products have very recently become more widely available for consumption, which was helped in part by the legalization of marijuana for either recreational or medical use by the majority of states in the United States [8]. There have subsequently been many identified cases of respiratory distress and lung injury in patients with recent vaping product use. These were first described and identified by the CDC in 2019, with 68 deaths reported thus far [1]. The mechanism of injury in acute lung injury after vaping remains poorly understood. A study in Belgium has shown that the propylene glycol and glycerol in vaping devices may be associated with decreased gas exchange and airway epithelial injury and that this injury pattern is not associated with nicotine [6]. Other recent literature suggests additives such as vitamin E acetate, diacetyl, and methanol in vaping products also may be implicated in this pattern of lung injury [4, 5]. It is extremely difficult to make the diagnosis of VALI because there are no readily available tests to confirm the presence of the disease. Testing BAL samples for vitamin E acetate, diacetyl, methanol are not available in most conventional laboratories, and the aforementioned studies identifying these compounds were performed in a CDC laboratory. One case report suggests that lipid-laden macrophages in BAL fluid samples may suggest VALI [9]. Another study of eight males with respiratory symptoms after vaping showed chest imaging findings of diffuse ground-glass opacities in all patients; however, this finding is not specific for VALI [10]. Diagnosis is supported mainly by recent (within 90 days) use of a vaping product; diffuse lung opacities visualized by radiography; exclusion of lung infection by sputum cultures, blood cultures, BAL, or other diagnostic criteria; and the absence of a likely alternative diagnosis [11, 12]. Given that our patient was young and immunocompetent, the likelihood that his presentation was due to an overwhelming rhinovirus or enterovirus pneumonia was very low [13]. It is important for physicians to remember that VALI is a diagnosis of exclusion and that they should manage these patients just as they would any other patient presenting with dyspnea: by using supportive care with oxygen therapy or other respiratory care management, administering fluids, and ruling out other life-threatening causes of dyspnea.

Once the diagnosis of VALI is highly suspected, it is important for physicians to remember just how potentially life-threatening the disease process is. Approximately 95% of patients presenting with VALI have required hospitalization [1]. Aside from supplemental oxygen, systemic glucocorticoids have been used to treat these patients; however, the efficacy has yet to be formally studied [9, 14]. Patients with respiratory failure or worsening respiratory status despite aggressive oxygen therapy may require intubation and/or ICU monitoring. Patients can only be considered for outpatient management if they do not have hypoxia (< 95%) on room air and have no signs of respiratory distress, no significant medical comorbidities that can contribute to worsening respiratory distress, and a strong social support system with close outpatient follow-up available [1].

## Conclusions

VALI is a disease that is still poorly understood overall and is currently being investigated by the CDC. In the meantime, physicians should consider vaping to be a public health emergency. Here we summarize the appropriate history, physical examination, appropriate workup, and therapies that physicians should be aware of in order to manage and treat patients presenting with VALI.

## Data Availability

Not applicable.

## Abbreviations

CDC: Centers for Disease Control and Prevention
CXR: Chest x-ray
CT: Computed tomography
E-cigarette: Electronic cigarette
ED: Emergency department
ICU: Intensive care unit
THC: Tetrahydrocannabinol
VALI: Vaping-associated lung injury

## References

[1] Centers for Disease Control and Prevention. Outbreak of lung injury associated with the use of e-cigarette, or vaping, products. https://www.cdc.gov/tobacco/basic_information/e-cigarettes/severe-lung-disease.html#epi-chart. Accessed 4 Aug 2020.

[2] Perrine CG, Pickens CM, Boehmer TK (2019). Characteristics of a multistate outbreak of lung injury associated with e-cigarette use or vaping: United States. Morb Mortal Wkly Rep.

[3] Ghinai I, Pray IW, Navon L (2019). E-cigarette product use, or vaping, among persons with associated lung injury: Illinois and Wisconsin. Morb Mortal Wkly Rep.

[4] Lal A, Mishra AK, Sahu KK (2020). Vitamin E acetate and e-cigarette or vaping product-associated lung injury (EVALI): an update. Am J Med.

[5] Kaur G, Muthumalage T, Rahman I (2018). Mechanisms of toxicity and biomarkers of flavoring and flavor enhancing chemicals in emerging tobacco and non-tobacco products. Toxicol Lett.

[6] Chaumont M, van de Borne P, Bernard A (2019). Fourth generation E-cigarette vaping induces transient lung inflammation and gas exchange disturbances: results from two randomized clinical trials. Am J Physiol Lung Cell Mol Physiol.

[7] Christiani DC (2020). Vaping-induced lung injury. N Engl J Med.

[8] National Conference of State Legislatures. State medical marijuana laws. Denver: National Conference of State Legislatures; 2019. http://www.ncsl.org/research/health/state-medical-marijuana-laws.aspx. Accessed 3 Sept 2020.

[9] Maddock SD, Cirulis MM, Callahan SJ (2019). Pulmonary lipid-laden macrophages and vaping [letter]. N Engl J Med.

[10] Mukhopadhyay S, Mehrad M, Dammert P (2019). Lung biopsy findings in severe pulmonary illness associated with e-cigarette use (vaping). Am J Clin Pathol.

[11] Schier JG, Meiman JG, Layden J (2019). Severe pulmonary disease associated with electronic-cigarette product use: interim guidance. Morb Mortal Wkly Rep.

[12] Layden JE, Ghinai I, Pray I (2020). Pulmonary illness related to e-cigarette use in Illinois and Wisconsin: preliminary report. N Engl J Med.

[13] Choi SH, Huh JW, Hong SB (2015). Clinical characteristics and outcomes of severe rhinovirus-associated pneumonia identified by bronchoscopic bronchoalveolar lavage in adults: comparison with severe influenza virus-associated pneumonia. J Clin Virol.

[14] Davidson K, Brancato A, Heetderks P (2019). Outbreak of electronic-cigarette-accociate acute lipoid pneumonia — North Carolina, July–August 2019. MMWR Morb Mortal Wkly Rep.

